# Working memory dysfunction in fibromyalgia is associated with genotypes of the catechol- O-methyltransferase gene: an event-related potential study

**DOI:** 10.1007/s00406-022-01488-4

**Published:** 2022-09-13

**Authors:** David Ferrera, Francisco Gómez-Esquer, Irene Peláez, Paloma Barjola, Roberto Fernandes-Magalhaes, Alberto Carpio, María Eugenia De Lahoz, María Carmen Martín-Buro, Francisco Mercado

**Affiliations:** 1 Department of Psychology, School of Health Sciences, Rey Juan Carlos University, Av. Atenas s/n. 28922, Alcorcón, Madrid, Spain; 2 Emerging Research Group of Anatomical, Molecular and Human Development Bases, Department of Basic Health Sciences, School of Health Sciences, Rey Juan Carlos University, Madrid, Spain

**Keywords:** COMT, P2, Working memory, Fibromyalgia, ERP

## Abstract

Recent findings have associated different COMT genotypes with working memory capacity in patients with fibromyalgia. Although it is thought that the COMT gene may influence neural correlates (P2 and P3 ERP components) underlying working memory impairment in this chronic-pain syndrome, it has not yet been explored. Therefore, the aim of the present research was to investigate the potential effect of the COMT gene in fibromyalgia patients on ERP working memory indices (P2 and P3 components). For this purpose, 102 participants (51 patients and 51 healthy control participants) took part in the experiment. Event-related potentials and behavioral responses were recorded while participants performed a spatial n-back task. Participants had to decide if the stimulus coincided or not in the same location as the one presented one (1-back condition) or two (2-back condition) trials before. Genotypes of the COMT gene were determined through a saliva sample from all participants. Present results significantly showed lower working memory performance (*p* < 0.05) in patients with fibromyalgia as compared to control participants (higher rate of errors and slower reaction times). At neural level, we found that patients exhibited enhanced frontocentral and parieto-occipital P2 amplitudes compared to control participants (*p* < 0.05). Interestingly, we also observed that only fibromyalgia patients carrying the Val/Val genotype of the COMT gene showed higher frontocentral P2 amplitudes than control participants (*p* < 0.05). Current results (behavioral outcomes and P2 amplitudes) confirmed the presence of an alteration in working memory functioning in fibromyalgia. The enhancement of frontocentral P2 could be reflecting that these patients would manifest an inefficient way of activating executive attention processes, in carriers of the Val/Val genotype of COMT. To our knowledge, the present findings are the first linking neural indices of working memory dysfunctions and COMT genotypes in fibromyalgia. Applying a subgroup of patient’s strategy based on this genetic marker could be useful to establish more tailored therapeutical approaches.

**Supplementary Information:**

The online version contains supplementary material available at 10.1007/s00406-022-01488-4.

## Introduction

Fibromyalgia is a chronic syndrome mainly characterized by non-specific widespread pain [1]. In addition to pain, patients commonly suffer from fatigue [2, 3], sleep problems [4, 5], affective and cognitive alterations [6–9], among other symptoms. In the last years, cognitive dysfunction in fibromyalgia has attracted growing research efforts. It has been suggested that these impairments are better explained by alterations in working memory subprocesses [10]. However, due to the symptoms’ complexity and heterogeneity in describing this chronic syndrome, findings on working memory impairments not always have been convergent [11–14]. Patient profiles or subgroups have been proposed as an alternative approach to overcome this controversy [15–18]. In this vein, genetic factors could become useful biomarkers for fibromyalgia.

Catechol-O-methyltransferase (COMT) gene is one of the possible candidates associated, at least partially, with inter-individual variability in physical, psychological or cognitive symptoms in fibromyalgia [3, 19–24]. This gene encodes an enzyme of the same name, which is involved in the degradation of catecholamines, such as dopamine, adrenaline or noradrenaline [25–27]. The COMT gene is located on chromosome 22 (22q11.2), spans about 27 Kb and has 6 exons and 2 promoters [28].The COMT gene locus contains dozen single nucleotide polymorphism (SNPs) [29] with minor allelic frequency greater than 1%. Most of them are located in noncoding regions and do not have an obvious potential for functional consequence. The most studied SNP in the COMT gene is the rs4680, also known as Val158Met. This polymorphism causes a substitution from valine (Val) to a methionine (Met) at amino acid position 158 [29, 30]. The Met allele is associated with low enzymatic activity and low protein stability [31]. The Met/Met genotype is associated with three to four times lower activity of the COMT enzyme than Val/Val genotype [32], whereas Met/Val seems to have intermediate levels of enzyme activity [33]. Briefly, the Met/Met genotype of the Val158Met polymorphism has been associated with an increase in tender points [19–22], the severity of fatigue [3], and depression or anxiety symptomatology in fibromyalgia [23, 34]. Complementarily, recent findings regarding cognitive impairment have indicated that patients with fibromyalgia bearing the Val/Val genotype showed a worsening in working memory tasks compared to healthy control participants carrying the same genotype [24]. However, evidence coming from the studies exploring the influence of COMT genotypes on cognitive functioning is far to be unequivocal [35–39]. In this regard, some authors have proposed the tonic/phasic dopamine hypothesis trying to solve such inconsistencies [36, 40]. In brief, this hypothesis proposes that the regulatory effect of dopamine on cortical (frontal) and subcortical regions (striatum) occurs through two processes: one phasic (derived from the transient release of high-amplitude dopamine) and the other tonic (characterized by long-lasting low-level dopamine release) [40, 41]. COMT genotypes seem to have different effects on the fronto-striatum dopaminergic network. Thus, whereas Met allele has been associated with tonic dopamine activity, leading to a better performance in tasks requiring cognitive stability, such as information maintenance [36, 38, 42], Val allele has been related to higher phasic dopamine levels [36]. This allele has been associated with a greater performance in tasks involving cognitive flexibility (updating or switching tasks) [38, 43, 44].

Given that the different working memory subprocesses (encoding, executive attention or updating [45, 46]) occur very fast, the use of techniques capable of identifying its neural time course, such as event-related potentials (ERPs), might be suitable for characterizing the dysfunctional neural mechanisms underlying working memory in fibromyalgia. This technique is derived from the electroencephalogram (EEG), which primarily records cortical electrical activity from postsynaptic potentials [47] of open-field neural structures [48, 49]. Along with the high temporal resolution offered by EEG, ERPs offer an increased signal-to-noise ratio (due to the averaging of many of the same or similar events) [47]. Furthermore, the positive or negative waves formed by the ERP appear to reflect different stages of sensory or cognitive processing [50], helping to understand such processes. Recently, some authors have reported lower parietal P2 amplitudes during a 2-back task [9], as a reflection of impairment in the encoding of information [51]. Furthermore, failures in context updating and replacement subprocesses of working memory have been also detected in fibromyalgia patients, as it was suggested by diminished parietal amplitudes in the P3 component [9]. This evidence could be consistent with the abnormal functioning the alterations of the frontoparietal neural network that have been previously reported in these chronic-pain patients [52].

It should be noted that several ERP components have shown a high heritability [53–55], however experimental evidence linking ERP data and genetic polymorphisms is still very scarce. Only a few studies have attempted to establish a relationship between P3 and different genotypes of the COMT gene in healthy participants and some pathologies, such as schizophrenia. Findings derived from these investigations were inconclusive about the significant influence of different COMT genotypes on P3 component [37, 56–59]. In addition, to our knowledge, no studies are exploring the relationship between COMT genotypes and P2 modulations, neither in healthy participants nor in chronic-pain patients.

Despite the high prevalence of cognitive impairment in fibromyalgia, the usefulness of ERPs methodologies for exploring the time course of working memory subprocesses and their close association with biological indices (i.e., COMT genotypes), the relationship between these three variables have not been explored up to date in this chronic-pain syndrome. Therefore, the aim of the present research was to investigate the potential effect of theVal158Met SNP of the COMT gene (genotypes: Met/Met, Met/Val and Val/Val) in fibromyalgia patients and healthy participants while ERP indices and behavioral measures were recorded in response to a spatial n-back task. Based on previous findings in patients with fibromyalgia, we expected that patients will exhibit both a lower task performance (higher reactions time and proportion of errors) and a decrement of P2 and P3 amplitudes. Furthermore, we expected to find a significant modulation of the COMT gene on the ERP indices of working memory.

## Materials and methods

### Participants

A group of 278 women (both healthy control participants and patients with fibromyalgia) underwent genotyping of the Val158Met/rs4680 polymorphism of the COMT gene. Of these participants, one hundred and fourteen right-handed participants took part in the experiment (57 patients with fibromyalgia and 57 healthy control participants). Finally, data from one hundred and two participants (51 patients with fibromyalgia and 51 controls participants) were analyzed, as it will be explained later. Patients fulfilled the 2016 American College of Rheumatology (ACR) diagnostic criteria for fibromyalgia [2]. They were recruited from the Fibromyalgia and Chronic Fatigue Syndrome Association of Comunidad de Madrid (AFINSYFACRO) and Fibromyalgia Association of Pinto (AFAP). Control participants were recruited among friends of patients and through both emailed and public advertisements located along with the School of health sciences of the Rey Juan Carlos University. All participants were aged between 35 and 68 years old. Patients with fibromyalgia and healthy control group were matched for age [F _(1,122)_ = 0.577, *p* = 0.449] and education level [χ^2^_(2)_ = 1.029, p = 0.598]. Participants had normal or corrected-to-normal eyesight and they had no history of psychiatric neurological or disorders that impaired cognitive functions. Moreover, none exhibited any disorder related to alcohol or drug abuse. Control participants did not suffer from any chronic-pain condition. Most patients with fibromyalgia were taking analgesics, benzodiazepines or antidepressants. Patients who were taking medications kept doing it because of both medical prescription and ethical considerations.

## Self-report measurements and psychological assessment

The Rey Juan Carlos University Research Ethics Board approved this study (ref: 0603201805018), and it followed all requirements from this Committee and the Declaration of Helsinki. Participants gave written informed consent for their involvement in the experiment. Once in the laboratory, different self-report instruments were administered to the participants just before starting the experimental session. All participants filled out State-trait anxiety inventory (STAI) [60]. The Beck Depression inventory (BDI) [61], the pain catastrophizing scale [62] and the Fear of pain questionnaire (FPQ-III) [63] were also administered. In addition, they completed a visual analog scale (VAS) for assessing both fatigue and pain during the previous week ranging from 10 (worse imaginable fatigue/pain) to 0 (no fatigue/pain at all). Finally, only patients with fibromyalgia had to complete the Fibromyalgia Impact Questionnaire (FIQ to evaluate their functional status and current health [64].

## Stimuli and experimental paradigm

**Fig. 1:**
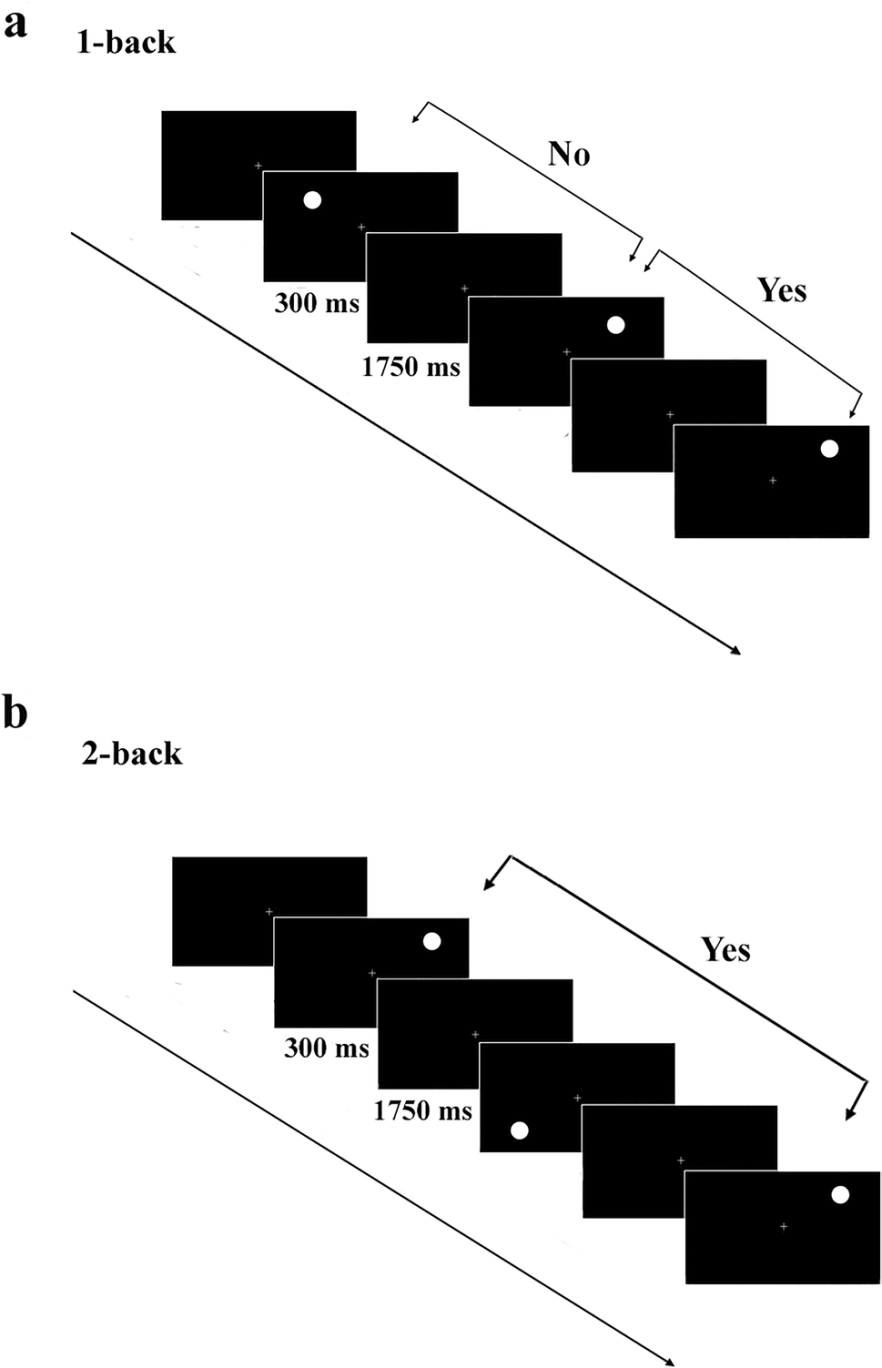
Schematic sequence of the spatial n-back task described in the main text. The participants were asked to give an affirmative answer if the dot presented on the screen was the same as the one presented one trials before **(a**:1-back**) **or two trails before (**b**: 2-back). In otherwise should give a negative answer. ITI = 2050 ms

Participants performed a spatial n-back task with two levels of cognitive load (1-back -low load- and 2-back -high load-). This task was originally used by Gevins and Cutillo [65] and later modified by Stokes and colleagues [66]. Commonly, n-back tasks consist of detecting whether a stimulus appearing on the screen is identical to the stimulus presented *n* times before. Specifically, in the paradigm here used, a white dot was peripherally presented at one of the four spatial localizations or quadrants in which the computer screen can be divided. Thus, participants were required to detect if the dot currently present on the screen was located in the same quadrant as the one that appeared in the previous trial (1-back condition) or twice before (2-back condition). All participants were instructed to continuously look at a small cross located in the center of the screen while the sequence of dots was presented. They completed the task sitting on a chair placed at 60 cm (eyes-screen distance) from the screen. Participants were asked to press with their right hand one button of a two keys device if the answer was affirmative (if the dot coincided in the same location as the one presented *n* trials before) and a different one if it was negative (if the dot was in different position as the one presented before). This task was configured in such a way that 50% of the answers were set to be negative. The order of stimulus presentation was semi-random, so that there were no more than three consecutive responses of the same type (affirmative or negative). Each dot (4.6 × 4.6 cm, 4.393° visual angle) was presented in white ink against a black background. It remained on the screen for 300 ms. The interval between dots was set at 2050 ms. The subject’s answer was recorded only if it came during the first 2000 ms that followed the dot onset, so any answer given past that time was considered as an omission. Both 1-back and 2-back tasks were divided into 4 blocks of 20 stimuli each to avoid fatigue interference. A total of 160 stimuli were presented (80 belonging to the 1-back and 80 for the 2-back condition). Figure 1 shows a schematic illustration of the experimental paradigm used.

## Electrophysiological recording

Brain electrical activity was recorded using an electrode cap (ElectroCap International) with 60 homogeneously distributed scalp electrodes. All these electrodes were referenced to mastoids. Vertical and horizontal eye movements were controlled through an electrooculographic (EOG) recording. Electrodes were placed infra- and supraorbitally to the left eye (vertical EOG). Another pair of electrodes was located at the outer canthus of each eye (horizontal EOG). A ground electrode was attached into an electrode cap between Fpz and Fz. All electrode impedances were kept below 5 kΩ. Online bandpass filters from 0.1 to 40 Hz (3 dB points for − 6 dB/octave roll-off) were applied for the recording amplifiers. Further, data were digitally filtered with a 30 Hz 24 dB/octave low-pass filter. Channels were continuously digitizing data at a sampling rate of 500 Hz throughout the entire recording session. Off-line pre-processing was performed using Brain Vision Analyzer software (Brain Products). The continuous recording was divided into 1000 ms epochs for each trial, beginning 200 ms before stimulus onset. EOG-artifact removal was conducted following the procedure described by Gratton and colleagues [67]. Baseline correction and EEG visual inspection were also carried out removing epochs with artifacts for further analyses. Data from twelve participants (6 fibromyalgia and 6 healthy control) were removed due to the high rate of artifacted trials (over 50%). Regarding the patient’s group, this artifact rejection procedure led to an admission trial average of 78.18% (mean = 62.55; SD = 10.07) for 1-back condition and 73.60% (mean = 58.88; SD = 8) for 2-back condition. The average of admitted trials for the control group was 73.30% (mean = 58.64; SD = 11.65) for 1-back condition and 71.07% (mean = 56.86; SD = 15.90) for 2-back condition. ERP averages were categorized according to each group of participants (patients with fibromyalgia and healthy control participants) and n-back condition (1-back and 2-back). Behavioral outcomes derived from the task performance [proportion of errors (PE) and reaction times (RT)] were also recorded and analyzed with respect to the group and cognitive load condition.

## COMT genotyping and control analysis

Genomic DNA was extracted from 5 ml of saliva using REALPURE Saliva RBMEG06 Kit (Durviz, Valencia, Spain) according to the manufacturer’s protocol.

The resulting DNA was diluted to 100–1000 ng/μl, using 1 × Tris‐EDTA (TE) buffer (Sigma‐Aldrich, Dorset, UK) and assessed for purity and concentration using a NanoDrop™ ND1000 Spectrophotometer (Thermo Fisher Scientific Inc., Hemel Hempstead, Hertfordshire, UK). COMT polymorphisms were genotyped by real-time polymerase chain reaction analysis using TaqMan® Predesigned SNP Genotyping Assays for rs4680 polymorphisms (Applied Biosystems). TaqMan® SNP Genotyping Assays use TaqMan® 5 ´‐nuclease chemistry for amplifying and detecting specific polymorphisms in purified genomic DNA samples. Each assay allows genotyping of individuals for a single nucleotide polymorphism (SNP). Each TaqMan® SNP genotyping assay contains: (A) Sequence-specific forward and reverse primers to amplify the polymorphic sequence of interest and (B) two TaqMan® minor groove binder (MGB) probes with non-fluorescent quenchers (NFQ): One VIC™‐labeled probe to detect Allele 1 sequence and One FAM™‐labeled probe to detect Allele 2 sequence. Amplification was carried out in ABI Prism 7000 Sequence Detection System (Thermo Fisher Scientific Inc., Hemel Hempstead, Hertfordshire, UK) in the Genomics and Flow Cytometry Unit of the Rey Juan Carlos University. All genotypes were determined twice.

Then, the chi-square (χ^2^) test was used to assess the distribution of the genotypes between patients and healthy control participants to meet the Hardy–Weinberg equilibrium (HWE).

## Detection and quantification of ERPs: P2 and P3 components

To identify and subsequently quantify P2 and P3 components of the ERPs, a principal component analysis (PCA) based on the covariance matrix was performed. This technique has been widely used in numerous studies for its advantages over analysis based on visual inspection of grand averages since it allows to avoiding subjectivity when selecting time windows based of EEG signal [68, 69]. Firstly, temporal PCA (tPCA) computes the covariance between all ERP time points, which tends to be high among those time points involved in the same component, and low between those belonging to different ERP components. Temporal factor (TF) score, the tPCA-derived parameter in which extracted temporal factors may be quantified, is linearly related to the amplitude of components (in this case, P2 and P3). The decision on the number of factors to extract was carried out through the application of the *scree test* [70]. Selected factors were Promax rotated as previously recommended [71].

## Analyses on ERPs, behavior and COMT genotypes

Because EEG signal overlapping can also occur at the spatial level, a spatial PCA (sPCA) on the TFs related to P2 and P3 was also carried out. Thus, while tPCA determines ERP components over time, sPCA separates them throughout the space (i.e., the scalp). Each spatial factor (SF) or scalp region would ideally reflect one of the concurrent neural processes (occurred at the same time) underlying each TF or ERP component (representing ideally each phase or subprocess of a given cognitive process). Therefore, this configuring and quantifying scalp regions system is preferable to an a priori subdivision into fixed scalp regions. In this case, SFs scores would reflect the amplitude of the whole spatial factor or electrode scalp region. This regional grouping was also determined through a covariance matrix-based sPCA and the decision on the number of factors to extract was based on the *scree test* as well. Extracted SFs were also submitted to Promax rotation.

Experimental effects were tested by computing a series of repeated measures ANOVAs for exploring the influence of group (two levels: patients with fibromyalgia and healthy participants), cognitive load condition (two levels: 1-back and 2-back) and COMT genotypes (three levels: Met/Met, Met/Val and Val/Val) on the factor scores corresponding to the P2 and P3 components. Thus, cognitive task load was included as the within-subject factor and the group of participants and COMT genotypes did so as between-subject factors. Regarding behavioral outcomes (PE and RTs), repeated measures ANOVAs were also computed including Group and COMT genotypes as the between-subject factors and task load as within-subject factor. Responses above 2000 ms or below 200 ms were detected and removed from the analyses. Greenhouse–Geisser (GG) correction was applied to adjust the degrees of freedom of the F ratios and to overcome sphericity violations. Post hoc comparisons to determine the significance of pairwise contrasts were performed using Bonferroni adjustment (α = 0.05) for controlling the Type I error rate. Effect sizes were computed using the partial eta-square (η^2^_p_) method.

Finally, several control analyses were also carried out to control the potential effect of benzodiazepines and antidepressants within the group of patients with fibromyalgia. We computed ANOVAs on both ERP components (P2 and P3) and behavioral measures (RT and PE), including fibromyalgia patients using and not using particular medications (benzodiazepines and antidepressants) as factor. All statistical analyses were done with SPSS package (v.25.0; SPSS Inc., Chicago; IL).

## Results

### COMT polymorphism frequencies

Statistical data related to genotypes and allele frequency distributions of the COMT gene considering each group of participants can be observed in Table 1. Frequency of the Val158Met polymorphism distribution fulfilled the HWE for both groups, healthy control participants (χ^2^ = 0.630; *p* = 0.427) and the patients with fibromyalgia (χ^2^ = 1.305; *p* = 0.253).

**Table 1:** Allele and genotype frequencies of the COMT gene in the patients with fibromyalgia and the healthy control participants

	Genotype frequencies *n* (%)			Allele frequencies	
Genotype	Control (*n*=57)	Fibromyalgia (*n*=57)		Control (*n*=57)	Fibromyalgia (*n*=57)
Val/Val	20 (35.1)	19 (33.34)	Val	0.43	0.44
Met/Val	25 (43.85)	24 (42.10)	Met	0.57	0.56
Met/Met	12 (21.05)	14 (24.56)			
*P* value HWE	0.427	0.253			

### Analyses on demographic, clinical and psychological data

To characterize the whole sample of participants, a series of ANOVAs were conducted. Results showed that patients with fibromyalgia scored significantly higher in BDI [F _(1,100)_ = 54.99, *p* = 0.001, η^2^_p_ = 0.355], STAI-State [F _(1,100)_ = 29.34, *p* = 0.001, η^2^_p_ = 0.227], STAI-Trait [F _(1,100)_ = 52.70, *p* = 0.001, η^2^_p_ = 0.345], fatigue VAS [F _(1,100)_ = 75.24, *p* = 0.001, η^2^_p_ = 0.429], pain VAS [F _(1,100)_ = 131.60, *p* = 0.001, η^2^_p_ = 0.568], PCS total [F _(1,100)_ = 44.48, *p* = 0.001, η^2^_p_ = 0.308] and their subscales (PCS rumination [F _(1,100)_ = 10.30, *p* = 0.002, η^2^_p_ = 0.093], PCS magnification [F _(1,100)_ = 3.25, *p* = 0.001, η^2^_p_ = 0.250], PCS helplessness [F _(1,100)_ = 76.18, *p* = 0.001, η^2^_p_ = 0.432]) and FPQ minor [F _(1,100)_ = 5.09, *p* = 0.048, η^2^_p_ = 0.048] than control participants. There were no significant differences in total [F _(1,100)_ = 0.55, *p* = 0.461], severe [F _(1,100)_ = 1.58, *p* = 0.211] or medical [F _(1,100)_ = 0.283, *p* = 0.596] fear of pain. Full details corresponding to socio-demographic, medication and clinical data for each group of participants are shown in Table 2.

**Table 2:** Means and standard deviations of age, level of trait and state anxiety, depression, pain, drug consumption and educational level for each group of participants. *P* values for each statistical contrast are also included

Clinical variables	Fibromyalgia patients	Healthy control	*P* value of F or χ^2^ test
Age	51.33 (7.45)	50.18 (8.77)	0.449
STAI			
STAI-state	48.27 (28.85)	22.27 (20.84)	**0.001**
STAI-trait	66.68 (28.66)	28.10 (24.88)	**0.001**
BDI	18.33 (11.42)	5.58 (4.48)	**0.001**
VAS pain	5.96 (2.12)	1.52 (1.76)	**0.001**
VAS fatigue	6.03 (2.43)	2.07 (2.17)	**0.001**
FIQ	53.29 (16.75)	−	−
PCS total	56.03 (25.11)	25.49 (20.96)	**0.001**
PCS rumination	48.53 (28.90)	31.21 (25.47)	**0.002**
PCS magnification	64.20 (24.71)	38.13 (20.75)	**0.001**
PCS helplessness	61.88 (24.38)	24.58 (18.34)	**0.001**
FPQ-III total	76.98 (24.87)	73.90 (16.20)	0.461
FPQ-III severe	35.64 (12.10)	33.21 (6.59)	0.211
FPQ-III minor	21.37 (7.85)	18.35 (5.43)	**0.048**
FPQ-III medical	23.07 (7.97)	22.31 (6.45)	0.596
Drug consumption			
Antidepressants (%)	49.0	2	**0.001**
Analgesics (%)	54.9	2	**0.001**
Benzodiazepines (%)	17.6	2	**0.008**
Others (%)	66.66	29.41	**0.001**
Educational level			
Elementary studies (%)	7.02	3.5	0.598
Middle level (%)	45.61	52.7	
Superior university studies (%)	47.37	43.8	

Statistically significant results are highlighted in bold

### Detection, spatio-temporal characterization, and quantification of P2 and P3

**Fig. 2:**
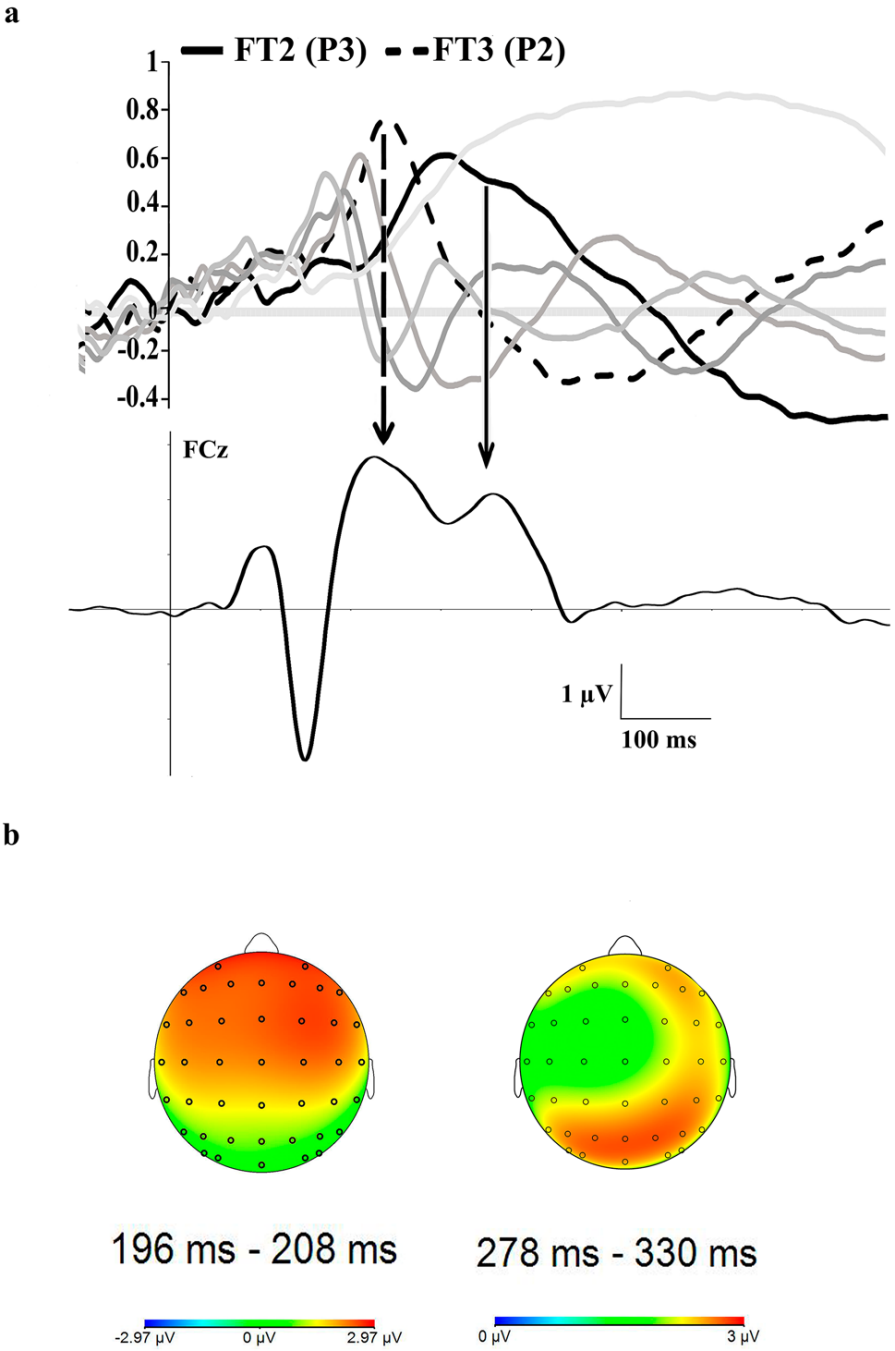
**a** tPCA: Factor loadings after Promax rotation. TF2 (P3 component) is highlighted in black continuous line and FT3 (P2 component) is drawn in black discontinuous line. **b** Scalp map distributions of the P2 and P3 components are also provided

Following the procedure described in the Method section, and after the application of the tPCA, five TFs were extracted from the ERPs (see Fig. 2 for the correspondence between P2 and P3 components and TFs derived from the tPCA). According to the factor peak latency and topography distribution, TF3 was associated with P2 and TF2 with P3 component (peaking around at 200 ms and 300 ms, respectively). Subsequently, after the application of sPCA, three spatial factors or scalp regions were extracted from each TF. P2: SF1 (parieto-occipital factor or region), SF2 (frontocentral factor or region) and SF3 (frontal factor or region); and P3: SF1 (parieto-occipital factor or region), SF2 (frontal factor or region) and SF3 (frontocentral factor or region). Full statistical data related to the rest of TFs (TF1, TF4, TF5 and TF6) are included in Supplementary Material 1.

### Experimental effects on the ERP components: P2 and P3

**Fig. 3:**
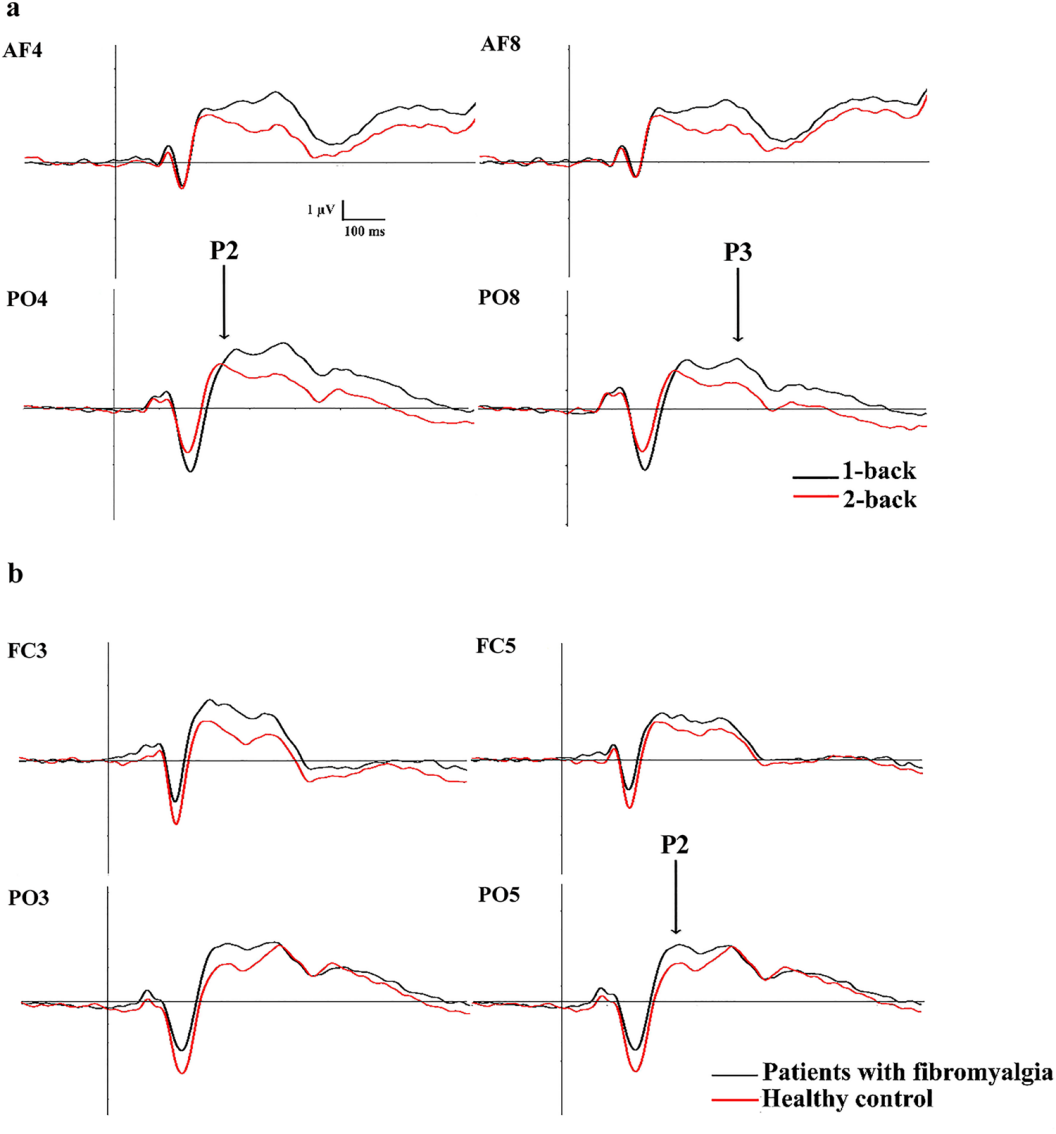
Grand averages representing **a** ERPs to both working memory load conditions (black lines represent 1-back condition and red lines 2-back condition) at frontal (AF4 and AF) and parieto-occipital electrodes (PO4 and PO8); and **b** ERPs for each group (red line represents brain responses to the healthy control group and black line shows the activity linked to patients with fibromyalgia) in frontocentral (FC3 and FC5) and parieto-occipital (PO3 and PO5) scalp sites

**Fig. 4:**
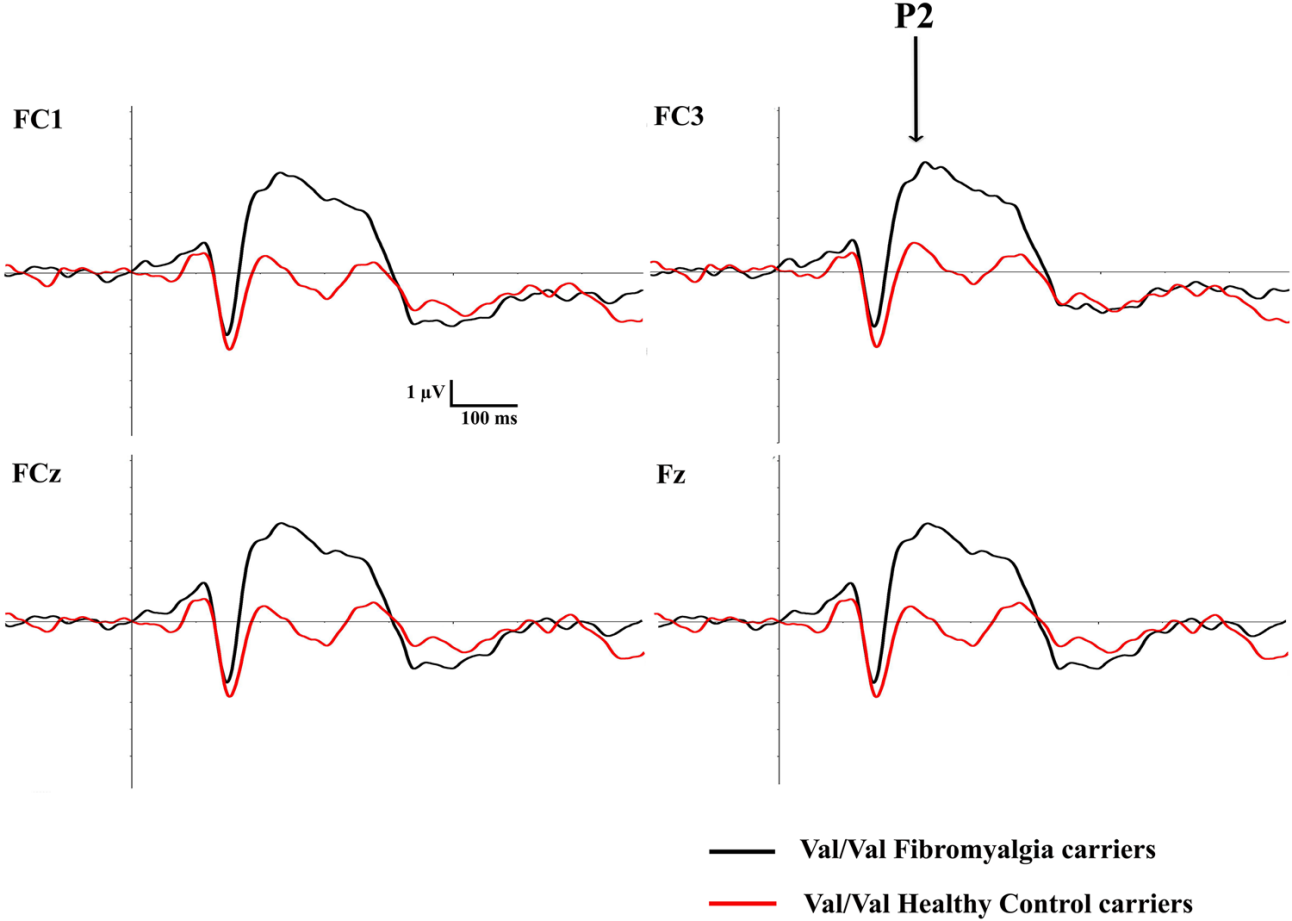
Grand averages showing brain responses (ERPs) for Val/Val genotype carriers in frontocentral electrode sites (FC1, FC3, FCz and Fz). Patients with fibromyalgia are represented in black line and healthy participants in red line

Grand averages where the most relevant experimental effects for P2 and P3 components can be seen in Figs. 3 and 4.

#### P2 analyses

Statistical analyses showed a main effect of working memory load in two different scalp regions of P2: parieto-occipital (SF1) [F _(1,96)_ = 12.540, *p* = 0.001, η^2^_p_ = 0.116] and frontal (SF3) [F _(1,96)_ = 13.337, *p* = 0.0001, η^2^_p_ = 0.122]. Both scalp regions presented lower amplitudes in P2 for 2-back task than 1-back condition (Fig. 3a). Furthermore, it was revealed a main effect of Group. Parieto-occipital [F _(1,96)_ = 4.193, *p* = 0.043, η^2^_*p*_ = 0.042] and frontocentral P2 amplitudes [F _(1,96)_ = 4.334, *p* = 0.04, η^2^_*p*_ = 0.043] were higher for patients with fibromyalgia as compared to healthy control participants (Fig. 3b). Finally, and more interestingly, we also observed an interaction effect between COMT gene by Group [F _(2,96)_ = 3.740, *p* = 0.027, η^2^_*p*_ = 0.072]. Specifically, post-hoc analyses revealed that patients with fibromyalgia carrying the Val/Val genotype showed greater P2 frontocentral amplitudes than healthy control participants carrying the same COMT genotype (*p* = 0.001; Fig. 4). No other significant interactions were found (see Table 3 for full information about this statistical contrast).

**Table 3:** Description of spatial factors belonging to TF3 (P2 ERP component) and TF2 (P3 ERP component), as well as their peak latency (ms) and distribution over the scalp. The results of the ANOVAs for each spatial factor are also shown, d.f.= degrees of freedom

Temporal factor	Peak	Scalp distribution	ANOVAs LOAD (d.f.=1,96)	ANOVAs COMT (d.f.=2,96)	ANOVAs GROUP (d.f.=1, 96)	ANOVAs COMT by GROUP (d.f.=2, 96)	ANOVAs LOAD by COMT (d.f.=2, 96)	ANOVAs LOAD by GROUP (d.f.=2, 96)	ANOVAs LOAD by GROUP by COMT (d.f.=2, 96)
TF3 (P2)	200ms	SF1(parieto-occipital) SF2 (frontocentral) SF3(frontal)	**F** **=** **12.540, *p*** **=** **0.001** F = 0.288, *p* = 0.593 **F** **=** **13.337, *p*** **=** **0.0001**	F = 1.823, *p* = 0.167 F = 0.371, *p* = 0.691 F = 1.233, *p* = 0.296	**F** **=** **4.193, *p*** **=** **0.043** **F** **=** **4.334, *p*** **=** **0.040** F = 1.095, *p* = 0.298	F = 2.944, *p* = 0.057 **F** **=** **3.740, *p*** **=** **0.027** F = 2.830, *p* = 0.064	F = 0.075, *p* = 0.928 F = 0.599, *p* = 0.551 F = 0.030, *p* = 0971	F = 2.287, *p* = 0.134 F = 2.558, *p* = 0.113 F = 0.034, *p* = 0.854	F = 0.749, *p* = 0.476 F = 1.052, *p* = 0.353 F = 0.024, *p* = 0.976
TF2 (P3)	300ms	SF1 (parieto-occipital) SF2 (frontal) SF3 (frontocentral)	**F** **=** **35.048, *p*** **= 0.0001** **F** **=** **13.100, *p*** **=** **0.0001** F = 1.371, *p* = 0.245	F = 1.732, *p* = 0.182 F = 1.068, *p* = 0.348 F = 0.505, *p* = 0.605	F = 0.003, *p* = 0.957 F = 0.017, *p* = 0.896 F = 0.698, *p* = 0.644	F = 0.430, *p* = 0.652 F = 0.576, *p* = 0.563 F = 0.588, *p* = 0.342	F = 0.766, *p* = 0.468 F = 0.583, *p* = 0.560 F = 1.033 *p* = 0.360	F = 0.085, *p* = 0.771 F = 0.144, *p* = 0.737 F = 0.207, *p* = 0.650	F = 0.452, *p* = 0.637 F = 0.146, *p* = 0.865 F = 1.125, *p* = 0.329

Statistically significant results are highlighted in bold

#### P3 analyses

ANOVAs revealed a main effect of working memory load for parieto-occipital (SF1) [F _(1,96)_ = 35.048, *p* = 0.0001, η^2^_*p*_ = 0.267] and frontal (SF2) P3 component [F _(1,96)_ = 13.100, *p* = 0.0001, η^2^_*p*_ = 0.120]. In both cases, P3 amplitudes for 2-back condition were lower than 1-back condition (Fig. 3a). Unexpectedly, the statistical analyses did not yield any significant relationship between COMT genotypes and P3 amplitudes at any scalp region. We found neither significant effect for the interaction between P3 by Group (full statistical details can be seen in Table 3).

### Behavioral data

Mean values for RT and PE associated with the performance on the n-back task (separated by group of participants), can be seen in Table 4. Repeated measures ANOVAs showed a main effect of task load for RT [F _(1,96)_ = 431.018, *p* = 0.0001, η^2^_*p*_ = 0.815], revealing that 2-back condition (mean = 729.20, SD = 12.18) generated slower RT than 1-back condition (mean = 496.86, SD = 8.91). Similarly, PE for 2-back condition (mean = 0.39, SD = 0.02) was higher than those associated with 1-back condition (mean = 0.10, SD = 0.01) [F _(1,96)_ = 269.287, *p* = 0.0001, η^2^_*p*_ = 0.733]. Furthermore, ANOVAs also yielded a main effect of Group for both RT [F _(1,96)_ = 5.709, *p* = 0.019, η^2^_*p*_ = 0.055] and PE [F _(1,96)_ = 8.069, *p* = 0.005, η^2^_*p*_ = 0.078]. In both cases, patients with fibromyalgia exhibited worse behavioral performance (i.e., higher PE (mean = 0.28, SD = 0.02) and RT (mean = 629.52, SD = 12.69)) than healthy control participants (PE (mean = 0.21, SD = 0.02) and RT (mean = 586.51, SD = 12.89), respectively) (Table 4). Finally, no significant behavioral effects were found with respect to COMT genotypes.

**Table 4:** Means and standard deviations (in parenthesis) of reaction times (RTs) and proportion of errors (PE) related to each COMT genotype (Val/Val, Met/Val and Met/Met). Data are showed separately for healthy control participants and patients with fibromyalgia

Load	COMT	Group	RT (ms)	Main effects on RT (*p*< 0.05)	PE	Main effects on PE (*p*<0.05)
1-back	Met/Met	Fibromyalgia	546.64 (95.187)		0.17 (0.16)	
		Healthy control	447.21 (65.93)		0.07 (0.04)	
	Met/Val	Fibromyalgia	524.65 (111.31)		0.13 (0.14)	
		Healthy control	476.5 (77.70)		0.09 (0.04)	
	Val/Val	Fibromyalgia	467.06 (86.80)	2-back > 1-back^1^	0.09 (0.06)	2-back > 1-back^1^
		Healthy control	458.97 (65.39)	Fibromyalgia > healthy control^2^	0.07 (0.04)	Fibromyalgia > healthy control^2^
2-back	Met/Met	Fibromyalgia	725.82 (121.44)		0.45 (0.23)	
		Healthy control	704.49 (113.925)		0.34 (0.15)	
	Met/Val	Fibromyalgia	778.02 (116.10)		0.46 (0.20)	
		Healthy control	721.53 (107.15)		0.37 (0.17)	
	Val/Val	Fibromyalgia	734.91 (122.51)		0.39 (0.19)	
		Healthy control	710.38 (132.68)		0.32 (0.16)	

^1^ Higher for 2-back than 1-back

^2^ Higher for fibromyalgia than healthy control

### Effects of medication on the ERP and behavioral data in fibromyalgia

Finally, statistical contrasts including the intake of psychotropics drugs by patients (i.e., benzodiazepines and antidepressants) did not reach statistical differences (*p* > 0.05) for any of the collected data (ERP or behavioral). Full statistical details can be observed in Table 5.

**Table 5:** P values related to medication effects (benzodiazepines and antidepressants) on behavior (proportion of errors and reaction times) and ERP activity (P2 and P3) after controlling the use of psychotropic drugs within the fibromyalgia group

	**Antidepressant ANOVAs**	**Benzodiazepines ANOVAs**
Electrophysiological data		
P2		
Parieto-occipital (SF1)	F _(1,49)_ = 0.098, p = 0.756	F _(1,49)_ = 0.535, p = 0.468
Frontocentral (SF2)	F _(1,49)_ = 0.125, p = 0725	F _(1,49)_ = 0.033, p = 0.857
Anterior (SF3)	F _(1,49)_ = 0.035, p = 0.852	F _(1,49)_ = 0.986, p = 0.326
P3		
Parieto-occipital (SF1)	F _(1,49)_ = 0.151, p = 0.699	F _(1,49)_ = 1.453, p = 0.234
Anterior (SF2)	F _(1,49)_ = 0.216, p = 0.644	F _(1,49)_ = 0.0001, p = 0.984
Frontocentral (SF3)	F _(1,49)_ = 0.211, p = 0.648	F _(1,49)_ = 0.596, p = 0.444
Behavioral data		
Proportion of errors	F _(1,49)_ = 2.426, p = 0.126	F _(1,49)_ = 0.263, p = 0.611
Reaction times	F _(1,49)_ = 3.208, p = 0.079	F _(1,49)_ = 0.023, p = 0.881

## Discussion

In the present research we applied a spatial n-back paradigm to explore how distinct indices of working memory capacity (ERPs and behavior) might be modulated by different genotypes of the COMT gene in patients with fibromyalgia. According to previous investigations, we observed that patients with fibromyalgia had a higher rate of errors and longer reaction times in the experimental task than healthy participants. There is extensive previous evidence that has consistently reported lower performance in fibromyalgia patients across various paradigms measuring working memory functioning (using both verbal and spatial tasks) [9, 24, 52, 72–74]. Therefore, working memory impairment in this chronic-pain syndrome seems to be robust [75] and highly relevant to general cognitive performance, as this process appears to underlie other mental functions [76]. At the neural level, current findings support the sensitivity of electrophysiological signals for revealing altered neural patterns in fibromyalgia [9, 77, 78]. Particularly, enhanced P2 amplitudes measured at parieto-occipital and frontocentral distributed scalp sites were identified for fibromyalgia patients compared to the healthy participants. To note, the most remarkable finding was that patients carrying Val/Val COMT genotype exhibited higher frontocentral P2 amplitudes compared to the healthy group. These neural modulations (peaking around 200 ms) were found regardless of the working memory load condition. Unexpectedly, any significant effect was found for P3 component when the influences of group (patients and control participants) or COMT genotype were tested. To our knowledge, the present findings, are the first linking ERP indices of working memory dysfunctions in chronic-pain syndromes, such as fibromyalgia, with different genotypes of the COMT gene.

As previously indicated, patients with fibromyalgia showed enhanced amplitudes of P2 component compared to healthy participants in two spatial regions (frontocentral and parieto-occipital) delimitated by spatial principal component analysis. Although there is still some debate about the significance of this cortical response, it has been argued that the posterior P2 component may represent memory encoding and recoding processing [51], whereas frontocentral P2 seem to be related to executive attention [79, 80]. Neuroimaging studies have indicated that a distinctive activation pattern involving prefrontal, but also inferior parietal cortices (frontoparietal memory network), might be underlying, at least partially, working memory impairment in fibromyalgia [52]. In the same line, parieto-occipital P2 modulations have been also recently reported when patients’ cognitive resources were involved in a verbal working memory task [9]. In that case, however, clear decrements in posterior P2 amplitudes were described. The question that now arises is what would be differentiating present findings from those obtained in previous research. Requirements linked to the n-back task (verbal versus visuo-spatial) seem to be different. Prior evidence has reported that whereas verbal working memory tasks would lead to the activation of right frontal regions, spatial tasks would activate left frontal ones [81–83]. Crucially, verbal items of a working memory task are associated with greater durability as mental representations, generating in turn, a higher degree of proactive interference than the interference generated in spatial tasks [84]. This may lead to verbal cognitive tasks to entail a higher load than spatial working memory paradigms making them more difficult. In this sense, the enhancement of parieto-occipital and frontocentral P2 amplitudes here detected might be reflecting the set-in motion of possible compensatory mechanisms [6, 85]. Given that spatial working memory tasks would demand a low degree of cognitive load, it would allow the implementation of such compensatory processes. Nevertheless, the higher cognitive load of verbal working memory task may prevent to activate them. It is thought that neurocognitive profile of patients with fibromyalgia has significant similarities with other clinical or subclinical populations. Particularly, these patients have shown an equivalent cognitive performance to that of older adults in working memory tasks [86]. Additionally, clear enhancements of P2 have been described in older people while they performed a cognitive task [87]. These data has been interpreted as a sign of the activation of compensatory neural mechanisms during low load tasks, but such cognitive effort cannot be triggered to deal with tasks involving a high degree of cognitive load [88–90]. The available data lead to think that this significant increase of cognitive resources in fibromyalgia would be, however, inefficiently allocated since behavioral performance remained significantly below that shown by healthy participants.‬ 

Particularly, the selective enhancement of frontocentral P2 amplitudes exhibited by patients with fibromyalgia carrying the Val/Val genotype deserves a detailed consideration. It has been observed that reduced synaptic levels of dopamine and reduced receptor activity associated with homozygous valine carriers seem to be a key factor that might contribute to a significantly poorer performance on working memory tasks in healthy people [91–94], but also in fibromyalgia patients [24]. Consequently, some investigations have proposed that such cognitive dysfunction may be related to the reduction in the efficiency of neural transmission that characterizes to valine carriers (i.e., higher neural activation but poor performance in cognitive tasks) [95]. This fact has been related to an increase of cortical noise in the activity of both prefrontal and frontocentral brain regions [56, 58, 96, 97]. Cortical noise would be manifested by random, less synchronized or less focused cortical activity [77] where excessive noise levels can lead to information processing difficulties [98]. Increased cortical noise during cognitive tasks has been also reported in patients with fibromyalgia [77] suggesting that it may be underling a more pronounced cognitive impairment. This fact, together with the generalized compensatory processing here detected in fibromyalgia, could contribute to explain the enhancement of neural indices (frontocental P2 amplitudes) in patients carrying Val/Val genotype of the COMT gene. As it was mentioned, frontal and frontocentral P2 activity has been associated with the activation of executive attention processes [79, 80]. Executive attention allows information to be actively maintained and manipulated [99], which makes this subprocess a crucial element for the correct performance in working memory tasks. Because this subprocess is closely linked to the capacity to maintain information for a given period of time, is thought that executive attention could be considered a subprocess that requires cognitive stability. Keeping this in mind, modulation of frontocentral P2 amplitudes (underlying executive attention) could fit the tonic/phasic hypothesis of dopamine. This theory postulates that the Met allele of the COMT gene could increase tonic activity (constant and slow firing neurons activity) whereas the Val allele would enhance phasic activity (neurons would have a transient, but large amplitude activity) [36, 38]. Neuropsychological studies have reported lower performance for valine carriers in tasks requiring cognitive stability (i.e., information maintenance) [36, 38, 42]. Thus, it could be thought that the influence of the COMT gene on the amplitude of the frontocentral P2 may be characterizing working memory dysfunction in fibromyalgia. On the other hand, the Met allele has been associated with higher reaction times and lower accuracy in tasks involving cognitive flexibility (updating or switching tasks) [38, 43, 44].

Surprisingly, available results did not reveal a relationship between COMT genotypes and P3 amplitudes. Unlike some prior investigations, we found no clear mediating role of the COMT gene (fibromyalgia patients carrying Val/Val genotype) on P3 amplitudes [57–59, 100]. Nevertheless, the present results must be interpreted considering different important factors. None of the of previous studies was focused on fibromyalgia syndrome (other patient’s sample were studied) and paradigms used (e.g., oddball task) involved other cognitive processes than those brought out by n-back tasks. Despite this, present electrophysiological data suggest that any influence of COMT gene on P3 amplitude, if exists, must be very subtle. Further research specifically focused on working memory dysfunction in fibromyalgia should be done (for instance, exploring the additive effects of other candidate genes related to dopamine regulation in neural networks) [101] to delimitate the potential influences of such genetic markers on different neural indices.

Some limitations should be considered with respect to the present findings. It has been pointed out that a single SNP may have small effects on the specific trait studied [102]. In this sense, the use of haplotypes can be a useful tool. It has been described that the use of several SNPs that form haplotypes may have a greater effect on gene function than nonsynonymous variations [30]. This haplotype-based strategy could shed light on some of the inconsistencies found in the present study. On the other hand, the selection of the rs4680 or Val158Met polymorphism of the COMT gene in this study is unlikely to cover all the genetic variations involving the different subprocesses of working memory. It has been suggested that genetic effects on working memory have an additive effect on dopamine regulation in prefrontal neural networks [101]. This additive effect would involve the influence of various genes related to dopamine regulation at different levels (dopamine receptors: DRD4, DRD1, DRD2 or dopamine transporters: DAT, among others) [101, 103–113]. It would be recommended that future investigation designs might consider the role of additional genes related to the regulation of dopaminergic transmission for exploring effects on cognitive performance in patients with fibromyalgia. Furthermore, future studies should be done to replicate these promising results using ERP and other EEG analysis methodologies, such as EEG oscillations or single-trial analyses in patients with fibromyalgia and other disorders with cognitive impairment. In this regard, it should be noted that the EEG represent a non-stationary signal [114], like occurs with almost real biological systems [115]. The use of different approaches such as the wavelet transform [116], time-varying autoregressive models [117], among others [118–120], would help to better analyze EEG signals and understand its temporal dynamics.

## Conclusions

In summary, present results suggest that COMT gene might influence ERP activity associated with working memory processing in fibromyalgia patients. Thus, patients with fibromyalgia carrying Val/Val genotype showed higher amplitudes of frontocentral P2 as compared to healthy valine carriers. It has been suggested that Val/Val genotype is associated with a high rate of cortical noise leading to a decrease in frontal efficiency to activate cognitive operations. This fact could be reflecting a specific impairment in executive attention process due to lower frontal efficiency in Val/Val fibromyalgia carriers. These promising data could help to better characterize working memory impairment in fibromyalgia, considering Val/Val genotype of the COMT gene as a biological marker useful to generate different patient profiles. Future research is needed to confirm present findings and measure up the possibility to establish more tailored treatments to deal with cognitive dysfunction in these chronic-pain patients.

## Supplementary Information

Below is the link to the electronic supplementary material.

Supplementary file1 (DOCX 14 KB)
