# Supplementary material for: Working memory dysfunction in fibromyalgia is associated with genotypes of the catechol- O-methyltransferase gene: an event-related potential study
Source: Eur Arch Psychiatry Clin Neurosci. 2022 Sep 13;273(1):25–40. doi: 10.1007/s00406-022-01488-4 (PMC9958168; doi:10.1007/s00406-022-01488-4)
Supplement: Supplementary file 1 — Supplementary file1 (DOCX 14 KB) [file 406_2022_1488_MOESM1_ESM.docx]

| **Temporal Factor** | **Peak** | **Associated component** | **Scalp Distribution** |
| --- | --- | --- | --- |
| TF1 | 550ms | LPC | SF1 parieto-occipital-occipital  SF2 frontocentral  SF3 frontal |
| TF4 | 150ms | N1 | SF1 parieto-occipital  SF2 anterior  SF3 central-parietal |
| TF5 | 120ms | P1 | SF1 parieto-occipital-occipital  SF2 anterior  SF3 frontal |
| TF6 | 100ms | N50 | SF1 parieto-occipital-occipital  SF2 frontocentral  SF3 frontal |

**Supplementary Material**. Temporal factor, peaks and their scalp distribution.
